# Gut microbial dysbiosis in individuals with Sjögren’s syndrome

**DOI:** 10.1186/s12934-020-01348-7

**Published:** 2020-04-15

**Authors:** Roberto Mendez, Arjun Watane, Monika Farhangi, Kara M. Cavuoto, Tom Leith, Shrish Budree, Anat Galor, Santanu Banerjee

**Affiliations:** 1 Department of Surgery, University of Miami, Miami, FL USA; 2 Bascom Palmer Eye Institute, University of Miami, Miami, FL USA; 3 Miami Veterans Administration Medical Center, Miami, FL USA; 4 OpenBiome, Cambridge, MA USA

**Keywords:** Sjögren’s syndrome, Dry eye, Gut microbiome, Dysbiosis

## Abstract

**Background:**

Autoimmune diseases have been associated with changes in the gut microbiome. In this study, the gut microbiome was evaluated in individuals with dry eye and bacterial compositions were correlated to dry eye (DE) measures. We prospectively included 13 individuals with who met full criteria for Sjögren’s (SDE) and 8 individuals with features of Sjögren’s but who did not meet full criteria (NDE) for a total of 21 cases as compared to 21 healthy controls. Stool was analyzed by 16S pyrosequencing, and associations between bacterial classes and DE symptoms and signs were examined.

**Results:**

Results showed that Firmicutes was the dominant phylum in the gut, comprising 40–60% of all phyla. On a phyla level, subjects with DE (SDE and NDE) had depletion of Firmicutes (1.1-fold) and an expansion of Proteobacteria (3.0-fold), Actinobacteria (1.7-fold), and Bacteroidetes (1.3-fold) compared to controls. Shannon’s diversity index showed no differences between groups with respect to the numbers of different operational taxonomic units (OTUs) encountered (diversity) and the instances these unique OTUs were sampled (evenness). On the other hand, Faith’s phylogenetic diversity showed increased diversity in cases vs controls, which reached significance when comparing SDE and controls (13.57 ± 0.89 and 10.96 ± 0.76, p = 0.02). Using Principle Co-ordinate Analysis, qualitative differences in microbial composition were noted with differential clustering of cases and controls. Dimensionality reduction and clustering of complex microbial data further showed differences between the three groups, with regard to microbial composition, association and clustering. Finally, differences in certain classes of bacteria were associated with DE symptoms and signs.

**Conclusions:**

In conclusion, individuals with DE had gut microbiome alterations as compared to healthy controls. Certain classes of bacteria were associated with DE measures.

## Background

Sjögren’s syndrome (Sjögren’s) is a chronic autoimmune disease characterized by oral and ocular dryness. It is a common autoimmune disorder, affecting 0.5–4% of the population, with more than 2 million Americans living with the disease [1]. Recently, there has been an interest in understanding interactions between gut bacteria and mucosal immunity in a number of eye diseases including Sjögren’s [2]. In a homeostatic state, commensal bacteria serve as a metabolically active organ and aid the host in a plethora of activities [3]. For example, many plant polysaccharides cannot be directly digested and are instead transformed by gut bacteria into short-chain fatty acids (SCFAs), like acetic acid and butyric acid [4]. Interestingly, some SCFAs enhance the death of effector T cells and promote proliferation of regulatory T cells in the intestine, and thus help suppress inflammation and the development of autoimmune disease [5]. On the other hand, abnormal alterations in the gut bacterial community (dysbiosis) can have negative effects on the host [6].

Individuals with autoimmune diseases have been found to have gut microbiome alterations compared to healthy controls, including individuals with spondylarthrosis, rheumatoid arthritis, Behçet’s, and Sjögren’s [7–10]. In Sjögren’s, greater relative abundances of *Pseudobutyrivibrio*, *Escherichia/Shigella* and *Streptococcus* and reduced relative abundances of *Bacteroides*, *Parabacteroides*, *Faecalibacterium,* and *Prevotella* were noted compared to controls. Reduced gut microbiome diversity was also found to correlate with overall disease severity [10]. The association between gut bacteria and autoimmune disease is likely a two-way street. On one hand, gut microbiome abnormalities can lead to systemic inflammation and, conversely, systemic inflammation can preferentially deplete beneficial gut bacteria and promote the growth of commensal bacteria with potential pathogenic properties [6, 11–14].

As there is limited data on gut microbial composition in Sjögren’s associated dry eye, we performed this study to evaluate the diversity, dimensionality and constituency of the gut microbiome in individuals with dry eye in a South Florida population and to correlate gut microbiome profiles to clinical parameters of disease. Understanding the interactions between intestinal biodiversity and the immune system will be fundamental in deciphering and treating the pathogenesis and causes of autoimmune diseases, including eye diseases [15].

## Results

### Study population

21 subjects were enrolled in the study (Table 1), 13 who met full Sjögren’s criteria (SDE) and 8 who did not (NDE). The mean age of the population was 60 years (range 33–71, standard deviation (SD) 8.8), 14 (67%) were female, 12 (57%) were white, and 8 (38%) were Hispanic. Comorbidities included diabetes (n = 2), hypothyroidism (n = 5), hypertension (n = 8), sleep apnea (n = 4), rheumatoid arthritis (n = 5), psoriatic arthritis (n = 1), systemic sclerosis (n = 1), and systemic lupus erythematosus (n = 1). In total, 8 subjects had a comorbid autoimmune disease, 3 in the SDE group and 5 in the NDE group. No significant differences were noted in demographics or comorbidities between the SDE and NDE groups (Table 1). The mean DEQ 5 was 11.6, mean OSDI was 41, and mean corneal staining was 7.2. Controls consisted of 21 individual samples provided by OpenBiome, who had no medical conditions or autoimmune diseases. The mean age of the controls was 26 (range 19–35, SD = 5.6) with all controls being male. Cases (SDE and NDE) (n = 21) were older than controls (59 vs 26, p = 0.07).

**Table 1:** Clinical characteristics of the study population

Variable	No. of patients		p-value	All cases (n = 21)
Demographics	SDE (n = 13)	NDE (n = 8)		
Age, years, mean ± SD (range)	58.8 ± 10.0 (33–71)	58.4 ± 7.0 (45–68)	0.90	58.7 ± 8.8 (33–71)
Gender, male, n (%)	4 (31%)	3 (38%)	0.76	7 (33%)
Race, white, n (%)	6 (46%)	6 (75%)	0.71	12 (57%)
Ethnicity, Hispanic, n (%)	3 (23%)	5 (63%)	0.40	8 (38%)
Smoking, n (%)	3 (23%)	1 (13%)	0.09	4 (19%)
Past, n (%)	2 (15%)	1 (13%)		3 (14%)
Current, n (%)	1 (8%)	0 (0%)		1 (5%)
Dry eye symptoms^a^				
DEQ5	10.8 ± 5.0 (0–17)	12.9 ± 4.6 (5–18)	0.36	11.6 ± 4.8 (0–18)
OSDI	37.5 ± 20.0 (12.5–83.3)	47.2 ± 26.5 (0–77.1)	0.35	41.2 ± 22.6 (0–83.3)
Dry eye signs^a^				
Inflammation via inflammadry	1.5 ± 1.2 (0–3)	1.0 ± 1.0 (0-3)	0.42	1.3 ± 1.1 (0–3)
Tear break up time	4.9 ± 2.1 (3–10)	7.0 ± 3.7 (2–13)	0.14	5.7 ± 2.9 (2–13)
Corneal staining	6.6 ± 2.7 (2–11)	8.3 ± 4.9 (1–14)	0.34	7.2 ± 3.6 (1–14)
Schirmer score	6.8 ± 2.4 (3–10)	10.1 ± 9.5 (1–25)	0.28	8.1 ± 6.1 (1–25)
Meibum quality	2.5 ± 0.9 (2–4)	2.0 ± 1.0 (2–3)	0.39	2.4 ± 0.9 (0–4)

*SDE* individuals who met full Sjögren’s criteria, *NDE* individuals who did not meet the full Sjögren’s criteria, *DEQ* *5* Dry Eye Questionnaire 5, *OSDI* ocular surface disease index, *SD* standard deviation (range)

*mean ± SD (range); ^a^more abnormal value between the two eyes

### Gut microbial landscape in cases compared to controls

**Fig. 1:**
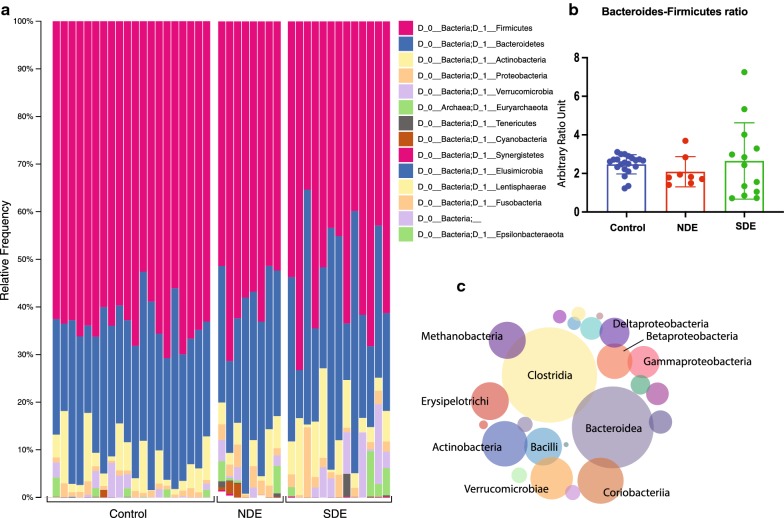
Overall distribution of bacterial phyla and classes in the gut microbiome of controls, individuals who met full Sjögren’s criteria (SDE) and those that did not (NDE). **a** All three study groups exhibit a Firmicutes-Bacteroidetes dominated microbiome, with significant presence of Actinobacteria and Proteobacteria. **b** Bacteroidetes-Firmicutes ratio shows an upward trend for the SDE group, but it is statistically insignificant. **c** Dominant representative bacterial classes among all study subjects across the three study groups

Firmicutes was the dominant phylum in the gut in all individuals, composing between 40 and 60% of all phyla, followed by Bacteroidetes. Cases (SDE + NDE) had a depletion of Firmicutes (1.1-fold), and an expansion of Proteobacteria (3.0-fold), Actinobacteria (1.7-fold) and Bacteroidetes (1.3-fold), compared to controls (Fig. 1a). There was no difference between cases and controls in terms of Bacteroides-Firmicutes ratio, although a lower ratio is considered a hallmark of inflammatory disease (Fig. 1b). While Clostridia, Bacteroidea and Actinobacteria were the dominant classes in all groups (Fig. 1c), Actinomycetaceae (3.6 fold, p = 0.01), Eggerthellaceae (6.2 fold, p = 0.001), Lactobacillaceae (8.8 fold, p = 0.02), Akkermanciaceae (4.7 fold, p = 0.04), Coriobacteriaceae (2.5 fold, p = 0.04) and Eubacteriaceae (7.4 fold, p = 0.02) had significantly increased abundance in cases compared to controls.

### Distance matrices show significant differences between case and control gut microbiota

**Fig. 2:**
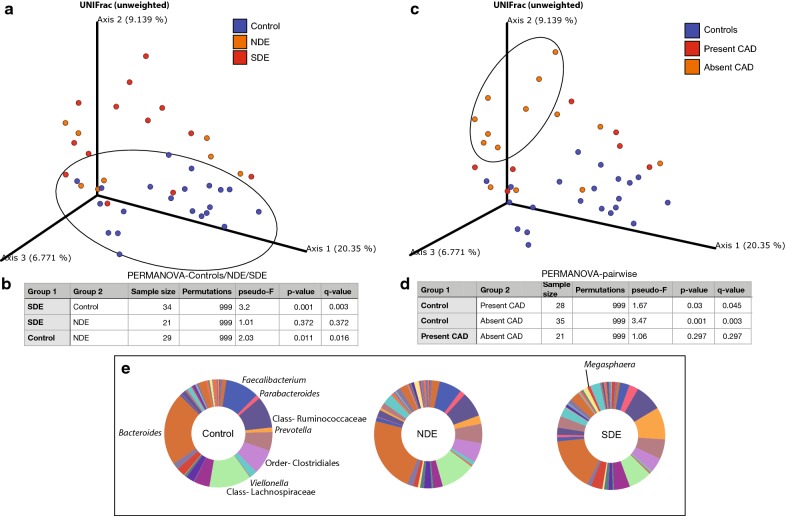
Microbial differences between controls and cases. **a** Unweighted-Unifrac Principal co-ordinate analysis (pCoA) at the OTU level showing the distribution of the control, NDE, and SDE groups. Controls (circled) cluster distinctly compared to cases. The FDR-adjusted p-value (q-value) when comparing age-related differences between cases and controls is 0.668. This separation in the groups by case definition suggests that microbial changes are driven by dry eye status and not age. **b** Pairwise PERMANOVA on the UniFrac distances (unweighted) showing significant differences between controls and each dry eye group (SDE and NDE). Compositional differences between SDE and NDE are not significant. **c** Unweighted-Unifrac Principal co-ordinate analysis (pCoA) at the OTU level showing the distribution of healthy controls and cases grouped by the presence or absence of a comorbid autoimmune disease. Individuals with dry eye and no comorbid autoimmune disease (CAD) (circled) cluster distinctly compared to controls. The separation in groups suggest that microbial changes are driven by dry eye and not co-morbid autoimmune disease. **d** Pairwise PERMANOVA on the UniFrac distances (unweighted) showing significant differences between controls and both the presence and absence of a CAD. **e** Major microbial components within controls, NDE, and SDE driving the significance above. Genera are italicized and upper hierarchical groups are labeled

Bray–Curtis principle co-ordinate analysis (pCoA; beta-diversity, (Fig. 2a) was used to qualitatively examine differences in microbial composition. There was a distinct clustering of controls compared to cases (blue, circled). When considering only individuals with overlapping ages in the study population, the groups still separated by DE status, suggesting that disease and not age was driving the effect. Next, unifrac distances were measured between the three groups and a pairwise PERMANOVA test was performed with false discovery rate correction (Fig. 2b; FDR; q-value). The same process was used to examine individuals with present versus absent comorbid autoimmune disease. The groups did not separate based on comorbid autoimmune status, suggesting that dry eye and not systemic autoimmune comorbidities were driving the effect (Fig. 2c and d).

**Fig. 3:**
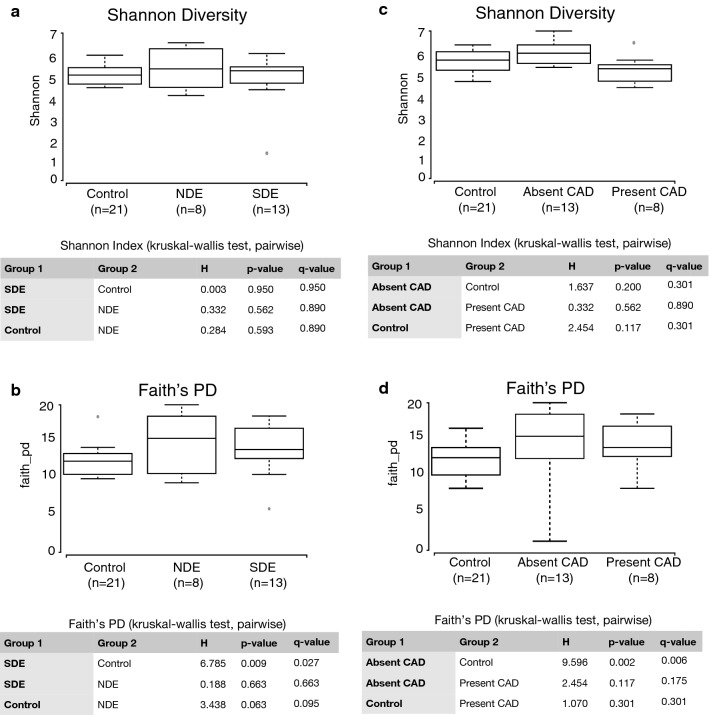
Microbial diversity between controls and dry eye cases, split into those who met full Sjögren’s-criteria (SDE) and those who did not (NDE) (**a**, **b**) and alternatively, classified based on the presence or absence of a comorbid autoimmune disease (CAD) (**c**, **d**). Among other α-diversity matrices shown in Table 3, two of the major indices Shannon’s H and Faith’s PD are displayed in the figure. For both parameters, Shannon’s diversity did not show any differences between the groups. Faith’s PD index showed significant differences between controls and SDE, and also between controls and the absence of a comorbid autoimmune disease

Both SDE and NDE groups independently exhibited significant compositional changes compared to controls. No difference was noted between the SDE and NDE groups. There was a decrease in genera *Faecalibacterium* and *Viellonella*, classes Ruminococcaceae and Lachnospiraceae, and orders Clostridiales and Bacteroides, when comparing controls to NDE and progressively to SDE. There was also an increase in the genera *Megasphaera*, *Parabacteroides* and *Prevotella* in SDE (Fig. 2e). These differences were major contributors to the significant differences seen in Figs. 2a–d, 3. *α*-Diversity matrices (Shannon and Faith) were unchanged between NDE and SDE (Fig. 3a and b respectively) and Shannon for Control and CAD (Fig. 3c), whereas the faith matrix was significantly changed between controls and ‘absent CAD’ as discussed later (Fig. 3d).

### Group-wise dimensionality reduction shows differential clustering between controls and both case groups

**Fig. 4:**
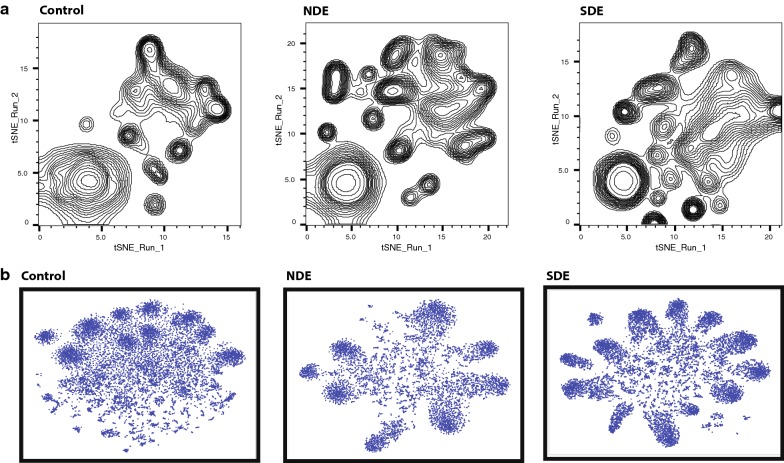
Dimensionality reduction of microbiome data and differential clustering within the three dry eye study groups. **a** t-Distributed Stochastic Embedding (t-SNE) implemented on group-wise OTU matrix demonstrate that control OTUs show a definite pattern of clustering that differs from those who met full Sjögren’s criteria (SDE) and those who did not (NDE). **b** Reference-independent deconvolution of bacterial sequences demonstrate distinct differences between SDE and NDE in terms of clustering and distribution of individual sequences. These differences are dramatic compared to Controls

We implemented non-linear dimensionality reduction on the microbiome data using both group-wise OTU matrix (Fig. 4a) and group-wise concatenated raw sequences (Fig. 4b). As mentioned in “Methods” section, these plots are phylogeny and sample ID agnostic and define a similar probability distribution of all OTUs within a group [16]. Similar parameters were used to generate a two-dimensional density plot for controls, NDE and SDE samples. As seen in Fig. 4a, control OTUs showed a definite pattern of clustering that differed from the NDE and SDE groups. The differences encompassed the appearance of distinct clusters with disappearance of others and the dissociation of coalesced clusters in cases compared to controls. These differences were more pronounced in the SDE group as compared to controls. These findings are indicative of an increase in relative abundance of distinct classes/genera of bacteria in both case groups (Fig. 2d). Differences in the population characteristics were even more evident when we did a reference-independent deconvolution of bacterial sequences as shown in Fig. 4b [17]. In controls, there was an amorphous distribution of sequences with 10 distinct major clusters, several minor clusters and numerous un-clustered sequences in the middle. This pattern differed dramatically in the case groups. In NDE, there was a major rearrangement of the sequences into major clusters, with a reduction in minor and un-clustered sequences. This pattern differed further in the SDE group, where more similar sequences rearranged into increased numbers of major clusters, at the expense of minor and un-clustered sequences.

### Bacterial association networks significantly change in cases compared to controls

**Fig. 5:**
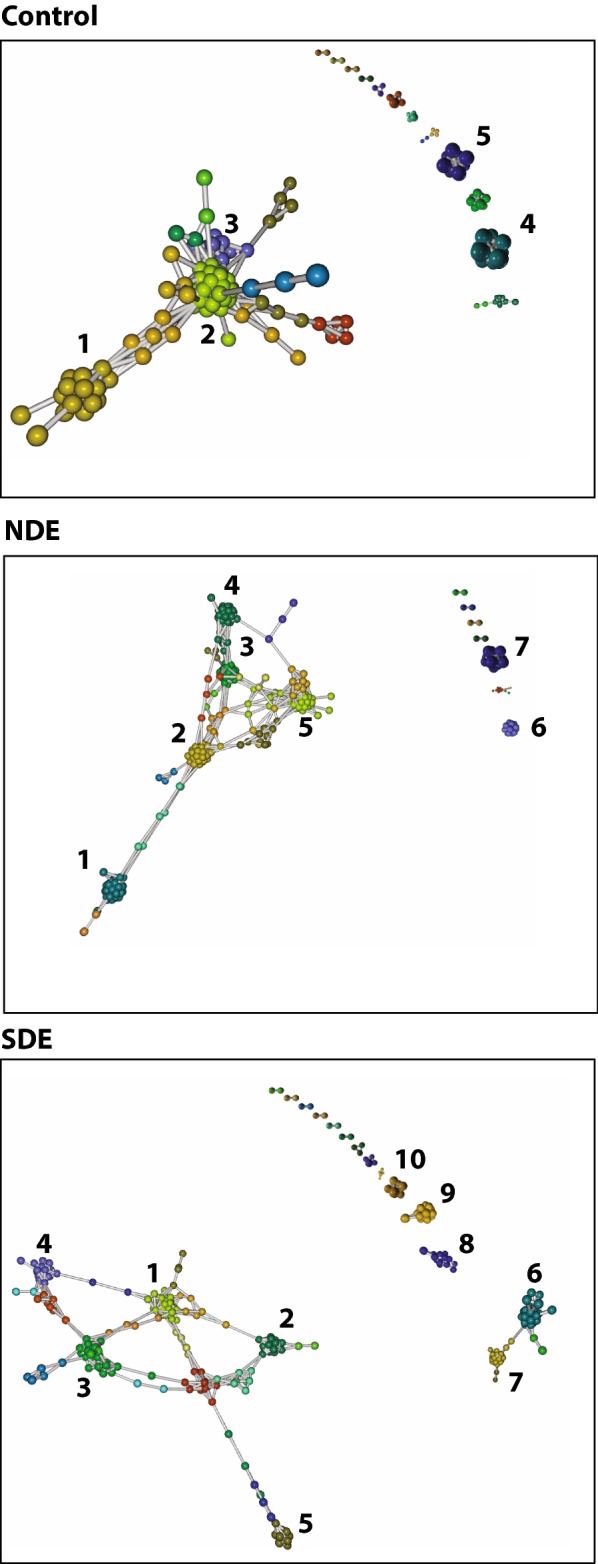
Markov Clustering algorithm and bacterial associations within the three study groups. As shown, control OTUs exhibit a single large super-cluster composed of 3 major clusters and several minor independent clusters. In comparison, in those who do not meet full Sjögren’s criteria (NDE), major constraints have been introduced into the network structure with the emergence of more clusters within the major super-cluster. In those who met full Sjögren’s criteria (SDE), these constraints seemed to be exacerbated, as the super-cluster stretched and expanded and new independent clusters emerged. Identities of the microbes comprising each cluster within the three groups is given in Supplementary sheet 1

Bacterial association was calculated using Markov Clustering Algorithm (MCL), which calculates correlations of vector abundance of OTUs within each group. This results in clustering of OTUs that are most likely to co-occur in a network, using correlation values as the distance matrix. For example, the higher the correlation value between 2 OTUs, the more likelihood that they would associate within a cluster. This also allows for each OTU to participate in multiple clusters. As shown in Fig. 5, control OTUs exhibited a single large super-cluster composed of 3 major clusters and several minor independent clusters. In NDE, major constraints were introduced into the network structure with the emergence of more clusters within the major super-cluster. In SDE, these constraints seemed to be exacerbated, as the super-cluster stretched and expanded and new independent clusters emerged. A comparison of the major clusters in control group to NDE showed a major rearrangement of bacterial association within its major clusters (Additional file 1: Figure S2 for controls, Additional file 1: Figure S3 for NDE and Additional file 1: Figure S4 for the SDE group). This included the increase in numbers of clusters, accompanied by addition of phyla representation in major clusters. Furthermore, within each phylum, it was evident that the genera representation differed compared to controls (e.g. expanded representation of the genus *Prevotella* in NDE clusters and the phylum Actinobacteria having different genera in each of the three groups). As evident from Fig. 5, SDE clusters have increased in numbers with major clusters composed of more diverse phyla with disparate genera representation.

### Dry eye parameters are associated with several bacterial classes

Finally, we evaluated the relationship between DE parameters and bacterial classes in the two case groups. As shown in Table 2, several bacterial classes exhibited an association to symptoms (DEQ 5, OSDI) and signs (ocular surface inflammation, corneal staining, tear production), when adjusting for age, gender, ethnicity and race in a multivariable model (Table 2).

**Table 2:** Multivariate analysis between clinical signs and gut microbial classes

Dry eye signs	Class	p^†^	Comparison with the literature
DEQ5	Methanobacteriaceae	< 0.01	↑ in RA and ulcerative Colitis [18]
	Bifidobacteriaceae	< 0.01	
	Eggerthellaceae	0.012	↓ in myasthenia gravis [19]
	Flavobacteriaceae	< 0.01	↓ in myasthenia gravis [19]
	Eubacteriaceae	<0.01	↑ in type 1 diabetes [20]
	Peptococcaceae	< 0.01	↓ in SLE [21]
	Ruminococcaceae	< 0.01	↓ in IBD and psoriasis [22]
	Erysipelotrichaceae	< 0.01	
	Leptotrichiaceae	< 0.01	
	Synergistaceae	< 0.01	
MMPWorse	Porphyromonadaceae	0.042	↑ in ankylosing spondylitis [23]
	Acidaminococcaceae	< 0.01	
OSDI	Rikenellaceae	0.046	↑ in ankylosing spondylitis [23]
Schirmer	Elusimicrobiaceae	< 0.01	
	Carnobacteriaceae	< 0.01	
	Clostridiaceae	< 0.01	↑ in SLE
	Clostridia Family XI	< 0.01	↑ in RA and IBD-arthritis [24]
	Clostridia Family XIII	< 0.01	
	Fusobacteriaceae	< 0.01	↑ IBD [21]
	Leptotrichiaceae	< 0.01	
	Akkermansiaceae	< 0.01	
Stainworse	Methanomassiliicoccaceae	0.028	
	Pasteurellaceae	< 0.01	↑ in myasthenia gravis [19]

*DEQ5* dry eye questionnaire 5, *OSDI* ocular surface disease Index, *RA* Rheumatoid arthritis, *IBD* inflammatory bowel disease, *SLE* systemic Lupus Erythematosus

^†^Multivariable analysis considered the effects of demographics (age, gender, race, ethnicity). Dry eye signs not listed in table (e.g. tear break up time) did not exhibit significant associations with gut microbial classes, when considering demographics

*For all dry eye signs, value from more severely affected eye used in the analysis

**Table 3:** Comparison between subjects with SDE vs NDE

Diversity indices/ Phylum/ratio	SDE Mean ± SD	NDE Mean ± SD	p-value
Chao1 diversity	116 ± 30	118 ± 35	0.88
Faith’s phylogenetic diversity	57.5 ± 10.7	60.7 ± 11.2	0.52
Shannon’s index	4.7 ± 0.64	4.9 ± 0.68	0.67
Observed OTU	891 ± 186	954 ± 155	0.44
Simpson diversity	0.076 ± 0.04	0.073 ± 0.04	0.90
Actinobacter	71 ± 70	45 ± 36	0.28
Bacteroides	332 ± 230	577 ± 744	0.39
Firmicutes	3502 ± 1898	4736 ± 1184	0.12
Proteobacteria	3.8 ± 10.0	1.3 ± 1.8	0.50
Firmicutes/Bacteroides	27 ± 58	30 ± 38	0.92

*SDE* individuals who met full Sjögren’s criteria, *NDE* individuals who did not meet full Sjögren’s criteria, O*TU* operational taxonomy unit, *SD* standard deviation

## Discussion

We demonstrated that individuals with dry eye, both those that met full Sjögren’s criteria (SDE) and those that did not (NDE), had gut microbiome alterations compared to controls. In our study, we found that cases had more diverse phyla with disparate genera representation compared to controls. These changes were found not to be driven by age or by the presence of a comorbid autoimmune disease. The microbial changes within Controls, NDE, and SDE included a decrease in genera *Faecalibacterium* and *Viellonella*, classes Ruminococcaceae and Lachnospiraceae, and orders Clostridiales and Bacteroides, and an increase in the genera *Megasphaera*, *Parabacteroides* and *Prevotella* from controls to NDE, and then from NDE to SDE. Similar changes in microbial association and networks have been shown to alter virulence and metabolic behavior of microbes in other disease models [25–27] and hence, assumes added importance in the role of host-microbiota interactions in health and disease. In addition, we expanded our exploration of composition beyond diversity to evaluate clustering patterns in disease, noting differences between controls, NDE and SDE with respect to pattern and clustering due to relative perturbations in bacterial populations. These changes in community behavior align with biological context, which demonstrate that compositional changes in microbiota due to disease is not a singular change. Changes in relative abundance of microbial species results in the imposition of constraints on co-occurrence network(s), resulting in induction/expulsion of other species, or complete rearrangement of networks [6]. We also found that several bacterial classes correlated with DE symptoms and signs, suggesting that the gut microbiome may impact disease severity (indirectly assessed via severity of DE metrics).

Our findings demonstrate both similarities and differences compared to prior data in Sjögren’s. In a study of 10 individuals with Sjögren’s, greater relative abundances of *Pseudobutyrivibrio, Escherichia/Shigella, Blautia* and *Streptococcus* and lower abundances of *Bacteroides*, *Parabacteroides*, *Faecalibacterium* and *Prevotella* were noted compared to 45 healthy controls identified from the Human Microbiome Project [10]. Similar to our population, controls were significantly younger than cases (27 ± 5 years old vs 59 ± 14 years old). A common finding in both studies was the presence of dysbiosis in Sjögren’s compared to controls, albeit with differences in bacterial signatures. Both studies noted a decrease in relative abundance of *Faecalibacterium* and *Bacteroides* in Sjögren’s, but in our study, we saw an increase in relative abundance of *Prevotella*, a bacteria implicated in rheumatoid arthritis [28, 29]. Another difference was in phylogenetic diversity, in which we found increased diversity in individuals with Sjögren’s compared to controls whereas the former study found a significant inverse correlation between diversity and disease severity (r = − 0.72, p = 0.01). Several differences must be considered when interpreting results between the two studies including differences in hypervariable region targets (V1–3 vs V4–5), curation status (2016 vs 2018) confidence interval (CI) of OTU database (97% vs 99%), controls (Human Microbiome Projects vs OpenBiome Stool Bank), geographical location (Texas vs Florida), and indices used (absolute OTU counts vs Faith’s PD). As a demonstration of how these differences can effect results, when we reanalyzed our data using the SILVA (v123) 97% CI database, the slight but significant increase in Faith’s PD in SDE vs controls was lost (Additional file 1: Figure S1). As Faith’s PD is a measure of number of nodes in a phylogenetic tree, it is understandable that values would change with a lower number of hits.

Our findings mirror changes noted in other autoimmune conditions, including ones related to Sjögren’s. Similar to our data, increased abundance of Actinobacteria [30], specifically *Eggerthella* and *Actinomyces, Prevotella copri* [28], *Lactobacillus* [31], and decreased abundance of *Faecalibacterium* [30], *Bacteroides* [8], *Lachnospiraceae* [28], and *Clostridiales* [28] were reported in individuals with rheumatoid arthritis as compared to controls. Interestingly, some of these dysbiotic signatures normalized with anti-inflammatory therapy [32]. In a similar manner, *Viellonella* was reduced in ankylosing spondylitis [33], *Ruminococcaceae* was reduced in inflammatory bowel disease and psoriasis [26], and *Megasphaera* was increased in primary biliary cirrhosis [34], all mirroring our findings in Sjögren’s. Beyond composition and not tested herein, bacterial metabolites of individuals with autoimmune disease has been found to differ from controls. For example, individuals with Behçet’s had less butyrate production in their gut compared to controls [9]. A similar finding was indirectly noted in Sjögren’s with a 50% decrease in relative abundance of OTUs classified to the high butyrate producer *Faecalibacterium prausnitzii* compared to controls [10].

These dysbiotic signatures may have a causal role in SDE. Inflammation is a hallmark of DE in individuals with and without Sjögren’s [35, 36], and it is well-established that the gut microbiome has an impact on inflammation and immunity [6, 11–14]. The commensal gut microbiome monitors mucosal immunity through the generation of anti-inflammatory regulatory T cells (Treg cells) and pro-inflammatory Th17 cells. The balance between these cells protects the mucosa from pathogenic microorganisms and limits excessive T cell responses via key mediators, including TGF-B, IL-6, retinoic acid and SCFA. For example, specific *Clostridia* species have been found to specifically induce Th17 cells in the small intestine and in extraintestinal sites during autoimmune inflammation. Other *Clostridia* clusters have been shown to induce Tregs and produce SCFAs to support Treg development [37]. In a similar manner, *Bacteroides* species can express polysaccharide A which suppresses Th17 inflammatory responses, allowing mucosal tolerance and subsequent colonization [37]. Putting this in context of our findings, reductions in commensals such as Clostridiales and *Bacteroides* may have an impact on the balance of Th17 and Treg cells, tipping the body towards autoimmunity.

Specific to the eye, altering the intestinal microbiome has been shown to influence eye disease. For example, CD25 knock out (KO) mice spontaneously develop DE and thus serve as a model of SDE. Germ-free CD25KO mice had a worse DE phenotype compared to CD25KO control mice, including increased lacrimal gland inflammation and IFN-ϫ producing T cells. Interestingly, recolonization of the gut microbiome improved the DE phenotype, with decreased lacrimal gland inflammation, IFN-ϫ producing T cells and corneal staining [38]. Similar findings have been seen in other mice models. Desiccating stress applied to the ocular surface with a fan in germ-free mice led to corneal staining and ocular surface inflammation, resulting in a worse DE phenotype compared to conventionally house mice [39]. In another experiment, antibiotics administered in addition to desiccating stress reduced Bacteroidetes and Firmicutes and increased Proteobacteria in the gut and concomitantly caused a more severe DE phenotype compared to desiccating stress alone [40]. These experiments reinforce the idea of a gut-eye axis and highlight the possibility of gut microbiome modulation and a therapeutic approach in DE.

Our findings should be interpreted bearing in mind our study limitations, which included a small, heterogenous population. The rationale for including both individuals who met full Sjögren’s criteria and who did not is that Sjögren’s is often diagnosed late in the disease course as the traditional markers, SSA and SSB, become positive years after disease initiation, if at all [41]. As such, many individuals with DE that have a specific profile (aqueous tear deficiency, early marker positivity, DE in the setting of an established autoimmune disease such as rheumatoid arthritis) are considered as having Sjögren’s-like DE but do not fit the ACR criteria for disease. In this study, we were interested in understanding gut microbiome profiles in both groups, although we acknowledge that the NDE group likely has a more heterogeneous makeup. Fortunately, as evident from our PCA plot, data from our diverse patient population is driven into a tight cluster, suggesting significant disease-mediated microbial changes in both groups, compared to controls. However, findings from our study will need to be replicated and expanded in larger populations. In addition, we chose controls provided by a stool bank (Openbiome) so as to compare our population to a well-phenotyped, healthy control group, which differed significantly in age and varied in gender. An issue with contemporary controls (e.g. age-matched veterans) is that other co-morbidities may affect microbiome health. As such, we modeled our work on prior studies, in which a similar approach also resulted in an age difference between cases to controls [10]. While age related differences in the gut microbiome have been noted when comparing very young children to adults, it seems that microbiome stabilizes to an adult-like composition by age 5 [42, 43]. Another limitation is that diet and consumption of probiotics were not considered in our evaluation, which may affect the composition of individuals’ gut microbiome [44]. In addition, 16s rRNA sequencing method has limited genera coverage, which limits a detailed study of the microbiome. Finally, our study did not measure metabolic products of bacteria, such as butyrate, which would be indicative of the function of the microbiome.

Despite these limitations, our study findings are important as they set the foundation for modulating the gut microbiome as a potential therapeutic approach in DE. There are several ways to modulate the gut microbiome, including dietary intake, probiotics and fecal microbial transplantation (FMT). For example, FMT was used to modulate the gut microbiome in Graft Versus Host Disease (GVHD), another condition associated with DE. In four individuals with GVHD, FMT increased abundances of the beneficial bacteria *Lactobacillus, Bacteroides, Bifidobacterium* and *Faecalibacterium,* and concomitantly improved gastrointestinal symptoms such as defecation consistency and frequency [45]. Future studies are needed to translate these findings to Sjögren’s-associated DE.

## Conclusion

The gut microbiome is altered in dry eye, and there are specific bacterial classes associated with dry eye signs and symptoms. This study sets the foundation for gut microbiome modulation as a potential therapeutic target for dry eye.

## Methods

### Study population

Individuals seen between November 2017 and February 2018 at the Miami Veterans Affairs (VA) Hospital or Bascom Palmer Eye Institute with dry eye were invited to participate. Individuals were split into two categories: (1) those who fulfilled the 2016 American College of Rheumatology criteria for Sjögren’s, having a total weighted-score of ≥ 4 from the sum of the following: (1) anti-SSA/Ro antibody positivity and focal lymphocytic sialadenitis with a focus score of ≥ 1 foci/4 mm^2^, each scoring 3; (2) an abnormal ocular staining score of ≥ 5, a Schirmer’s test result of ≤ 5 mm/5 min, and an unstimulated salivary flow rate of ≤ 0.1 ml/minute, each scoring 1 [46] and (2) those with dry eye (DE) symptoms and features of Sjögren’s but whom did not meet full criteria. This included individuals with (1) ≥ 1 early Sjögren’s marker positivity [47]; (2) aqueous tear deficiency (ATD) defined as Schirmer score with anesthesia ≤ 5 mm in either eye; or (3) an autoimmune disease (e.g. rheumatoid arthritis). We first combined the two groups and then examined each group individually as compared to controls. Given the concern that systemic autoimmune disease may affect the composition of the gut microbiome, in a secondary analysis, we split cases into two groups based on the presence or absence of a comorbid autoimmune disease (e.g. rheumatoid arthritis).

### Ethical approval

The Miami VA and University of Miami Institutional Review Boards (IRB) approved the prospective evaluation of patients. Informed consent was obtained from all subjects and the study was adherent with the principles of the Declaration of Helsinki. The IRB number was 20170733.

### Clinical metrics

Demographic information for each participant was collected including age, gender, race, ethnicity, past ocular and medical history and current medications.

### DE symptoms

Participants completed two standardized DE symptom questionnaires: the Dry Eye Questionnaire 5 (DEQ 5) [48] (score 0–22) and the Ocular Surface Disease Index (OSDI) [49] (score 0–100).

### DE signs

Participants underwent a complete ocular surface exam of both eyes in the following order:

1. Ocular surface inflammation via matrix metalloproteinase (MMP) 9 levels (Inflammadry, Quantel, San Diego, CA) [50] graded based on the intensity of the pink line (0 = no line, 1 = faint pink line, 2 = pink line, 3 = intense pink line).
2. Tear breakup time (TBUT) using fluorescein stain measured three times in each eye and averaged.
3. Corneal staining using fluorescein stain evaluated using the National Eye Institute (NEI) scoring scale which assesses 5 areas of the cornea on a 0–3 scale with a total score generated by summing the 5 section scores [51].
4. Basal tear production after anesthesia placement (measured in mm at 5 min) using Schirmer’s strips.
5. Meibum quality evaluated after expression with intermediate pressure applied to the lower eyelid (0 = clear; 1 = cloudy; 2 = granular; 3 = toothpaste; 4 = no meibum extracted).

### Stool collection and analysis

All subjects were given a stool collection kit for at home collection. Stool samples were collected and placed in a glycerol suspension, homogenized, and sent to OpenBiome (Cambridge, MA). Specimens were then frozen at − 80 °C until analysis. Total DNA was isolated using Power-soil/fecal DNA isolation kit (Mo-Bio, Germantown, MD) as per manufacturer’s specifications. All samples were quantified using the Qubit^®^ Quant-iT dsDNA Broad- Range Kit (Life Technologies, Grand Island, NY) to ensure that they met minimum concentration and mass of DNA [52]. To enrich the sample for the bacterial 16S V4 rDNA region, DNA was amplified using fusion primers designed against the surrounding conserved regions that are tailed with sequences to incorporate flow cell adapters and indexing barcodes (Illumina, San Diego, CA). Each sample was PCR amplified with two differently barcoded V4–V5 fusion primers and were advanced for pooling and sequencing. For each sample, amplified products were concentrated using a solid-phase reversible immobilization method for the purification of PCR products and quantified by electrophoresis using an (Agilent, Santa Clara, CA) 2100 Bioanalyzer. The pooled 16S V4V5-enriched, amplified, barcoded samples were loaded into the MiSeq cartridge (Illumina Inc, San Diego, CA), and then onto the instrument along with the flow cell. After cluster formation on the MiSeq Instrument (Illumina, San Diego, CA), the amplicons were sequenced for 250 cycles with custom primers designed for paired-end sequencing.

In addition to patient samples, reagent controls were supplied in triplicate as background. Samples producing amplicons at later cycles compared to majority of samples were concentrated using Agencourt AMPureXP beads (Beckman Coulter, Indianapolis, IN). All samples were sequenced together after barcode-normalization subsequent to a preliminary sequencing run.

Using QIIME 2.0 [53], sequences were quality filtered and de-multiplexed using exact matches to the supplied DNA barcodes and primers. Resulting sequences were then searched against the SILVA database (v123) and clustered at 99% to obtain phylogenetic identities.

### Statistical analysis

OTU tables were rarefied to the sample containing the lowest number of sequences in each analysis. QIIME 2.0 was used to calculate alpha diversity and to summarize taxa [53]. Descriptive statistics were used to describe relative compositions of bacteria on phyla, genera and class levels. Data were analyzed for significance (p ≤ 0.05) by 2 tailed student t and Mann–Whitney U tests (GraphPad Prism 8).

### Principal coordinate analysis

UNIFrac principal coordinate analysis (PCA) was performed using observation ID (OTU) level. The PERMANOVA test was utilized to find significant whole microbiome differences among discrete categorical or continuous variables with randomization/Monte Carlo permutation test (with Bonferroni correction). The fraction of permutations with greater distinction among categories (larger cross-category differences) than that observed with the non-permuted data was reported as the p-value. False discovery rate (FDR) corrected p-value (q-value) < 0.05 was considered significant across groups.

### Comparison of diversity indices

α-diversity matrices were compared between groups using Kruskal–Wallis pairwise rank tests or its variant, the Mann–Whitney U test.

### Dimensionality reduction and bacterial association analysis

We utilized both sequence-based and OTU-matrix dimensionality reduction and clustering algorithms. Compared to Qiime-derived PCA, which is done sample-wise, these algorithms are identity agnostic and decipher qualitative association (and disassociations) between experimental groups. The following methods were used:

- t-SNE (t-Distributed Stochastic Embedding): t-SNE algorithm was implemented using group-wise OTU matrix with SeqGeq 1.5.0 software (FloJo LLC, Ashland, OR). t-SNE plots were generated with a perplexity value of 30 and 1000 iterations.
- Reference-independent binning: This algorithm performs a reference-independent deconvolution of metagenomic sequences to reduce non-linear dimensionality of the samples. We used the java implementation of VizBin for this analysis [17]. We used a minimum contig length of 200 bases, k-mer length of 5, perplexity 30 with 1000 iterations for this study. Both individual samples and concatenated groups were analyzed.
- Bacterial association and clustering: We used Graphia software (Edinburgh, UK) and its implementation of Markov Clustering algorithm (MCL) [54]. Using our group-wise OTU matrix, MCL looked for cluster structures using mathematical bootstrapping. While this method is still agnostic to phylogenetic hierarchy in the matrix, we used the hierarchy as identifying markers to understand the bacterial clusters and changes in those clusters within the 3 study groups. MCL used the stochastic flow of the matrix to decipher the distances between the OTUs at equilibrium, thereby generating a cluster map by using correlation scores as distance. For generating the cluster, nodes scoring above a Pearson correlation value of 0.85 were used.

### Correlations between bacterial classes and clinical measures

Multivariable linear regression analyses were performed to evaluate associations between bacteria classes and DE measures, considering demographics (age, gender, race, and ethnicity) in the model.

## Supplementary information

**Additional file 1:** Figure S1: Faith's PD when calculated between Controls, NDE and SDE at 97CI (A) and 99CI(B). Figure S2: Constituents of identified clusters in main Figure 5a (Controls). Figure S3: Constituents of identified clusters in main Figure 5b (NDE). Figure S4: Constituents of identified clusters in main Figure 5c (SDE).

## Data Availability

The datasets used and/or analysed during the current study are available from the corresponding author on reasonable request.
