# Supplementary material for: Gut microbial dysbiosis in individuals with Sjögren’s syndrome
Source: Microb Cell Fact. 2020 Apr 15;19:90. doi: 10.1186/s12934-020-01348-7 (PMC7158097; doi:10.1186/s12934-020-01348-7)
Supplement: Supplementary file 1 — Additional file 1: Figure S1: Faith's PD when calculated between Controls, NDE and SDE at 97CI (A) and 99CI(B). Figure S2: Constituents of identified clusters in main Figure 5a (Controls). Figure S3: Constituents of identified clusters in main Figure 5b (NDE). Figure S4: Constituents of identified clusters in main Figure 5c (SDE). [file 12934_2020_1348_MOESM1_ESM.pdf]

Additional Figure

Figure S1

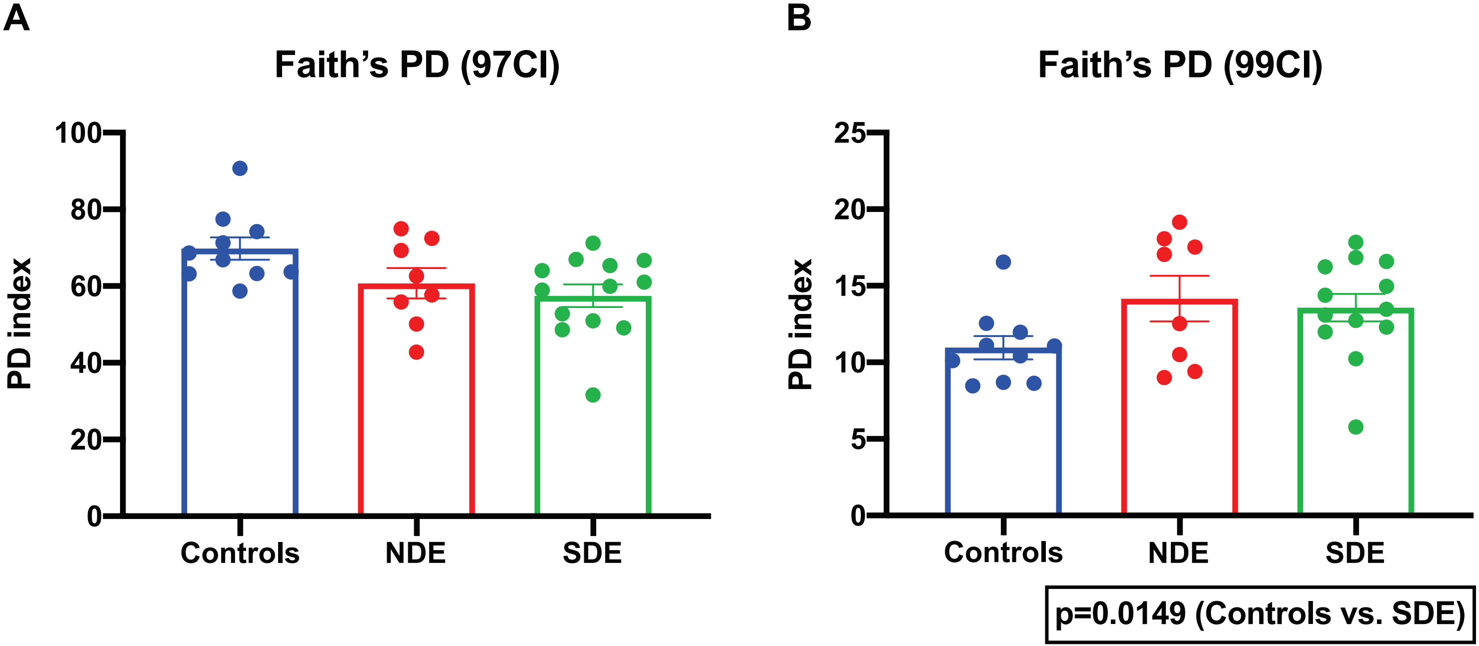

Figure S2

Table 1

	Cluster 1			Cluster 2			Cluster 3			Cluster 4			Cluster 5	
	Phylum	Species		Phylum	Species		Phylum	Species		Phylum	Species		Phylum	Species
	Actinobacteria	Bifidobacterium		Euryarchaeota	Methanobrevibacter		Actinobacteria	Slackia		Actinobacteria	Raoultibacter		Actinobacteria	Bifidobacterium
	Bacteroidetes	Bacteroides		Actinobacteria	Varibaculum		Bacteroidetes	Coprobacter		Bacteroidetes	Odoribacter			Eggerthella
		Paraprevotella			Collinsella			Odoribacter		Firmicutes	Lachnospiraceae ND3007 group		Firmicutes	Blautia
		Prevotella			Gordonibacter			uncultured organism			Tyzzerella 3			GCA-900066575
		Alistipes		Bacteroidetes	Coprobacter			Alistipes			Fournierella			Lachnospiraceae ND3007 group
	Firmicutes	Lactobacillus			Odoribacter		Cyanobacteria	Clostridium sp. CAG:306			[Eubacterium] coprostanoligenes group			[Ruminococcus] gnavus group
		Streptococcus			uncultured bacterium						Faecalitalea			[Eubacterium] coprostanoligenes group
		Family XIII AD3011 group			uncultured organism		Firmicutes	Christensenellaceae R-7 group			Holdemanella			Faecalitalea
		Eubacterium nodatum group						Christensenellaceae R-7 group			Phascolarctobacterium			Megasphaera
		Lachnoclostridium			Alistipes			uncultured						
		Lachnospiraceae NK4A136 group		Cyanobacteria	Clostridium sp. CAG:306					Proteobacteria	Parasutterella			
		Terrisporobacter						gut metagenome						
		CAG-352		Firmicutes	Christensenellaceae R-7 group			uncultured bacterium						
		DTU089			Christensenellaceae R-7 group			uncultured organism						
		Oscillibacter			uncultured			Defluviitaleaceae UCG-011						
		Allisonella						[Eubacterium] brachy group						
	Proteobacteria				uncultured bacterium			Blautia						
		Desulfovibrio			uncultured organism			Coprococcus 3						
		uncultured						Lachnospiraceae FCS020 group						
		Sutterella			Peptoniphilus			Lachnospiraceae ND3007 group						
					Blautia			Marvinbryantia						
					Blautia			[Eubacterium] ruminantium group						
					Coprococcus 3			uncultured						
					Lachnospiraceae FCS020 group									
					Marvinbryantia			Peptococcus						
					uncultured			uncultured						
					Peptococcus			Hydrogenoanaerobacterium						
					uncultured			Intestinimonas						
					uncultured			Oscillibacter						
					Butyricicoccus			Ruminiclostridium 5						
					Hydrogenoanaerobacterium			Ruminiclostridium 9						
					Intestinimonas			Ruminococcaceae UCG-002						
					Oscillibacter			Ruminococcaceae UCG-002						
					Ruminiclostridium 5			Ruminococcaceae UCG-005						
					Ruminiclostridium 9			Ruminococcaceae UCG-005						
					Ruminococcaceae UCG-002			Ruminococcaceae UCG-005						
					Ruminococcaceae UCG-002			Ruminococcaceae UCG-010						
					Ruminococcaceae UCG-005			Subdoligranulum						
					Ruminococcaceae UCG-005			[Eubacterium] coprostanoligenes group						
					Ruminococcaceae UCG-010									
					Ruminococcaceae UCG-010			Candidatus Stoquefichus						
					Subdoligranulum			[Clostridium] innocuum group						
					[Eubacterium] coprostanoligenes group			uncultured						
					uncultured									
								Victivallis						
								Desulfovibrio						
					Candidatus Stoquefichus									
					[Clostridium] innocuum group			Escherichia-Shigella						
					uncultured									
				Lentisphaerae	Victivallis									
				Proteobacteria	Desulfovibrio									
							Lentisphaerae							
					Escherichia-Shigella		Proteobacteria							
				Tenericutes	gut metagenome									
				Verrucomicrobia	Akkermansia									

Figure S3

Table 1

	Cluster 1		Cluster 2		Cluster 3		Cluster 4		Cluster 5		Cluster 6		Cluster 7		
	Phylum	Species	Phylum	Species	Phylum	Species	Phylum	Species	Phylum	Species	Phylum	Species	Phylum	Species	
	Actinobacteria	Eggerthella	Euryarchaeota	Methanobrevibacter	Euryarchaeota	Methanomassiliicoccus	Actinobacteria	Raoultibacter	Actinobacteria	Bifidobacterium	Epsilonbacteraeota	Campylobacter	Actinobacteria	Enorma	
		Gordonibacter	Actinobacteria	Actinomyces	Actinobacteria	Atopobium		uncultured	Bacteroidetes	Bacteroides	Firmicutes	Gemella		uncultured	
		uncultured		Bifidobacterium		Enterorhabdus		CHKCI002		uncultured				CHKCI002	
	Bacteroidetes	Paraprevotella		uncultured		Paraeggerthella				Butyricimonas		Streptococcus			
		Prevotella 2		Gordonibacter	Actinobacteria	Slackia	Bacteroidetes	Sanguibacteroides		Odoribacter		Streptococcus	Epsilonbacteraeota	Campylobacter	
		Prevotella 7		Paraeggerthella	Bacteroidetes	Bacteroides		Paraprevotella	Firmicutes	Enterococcus		Eisenbergiella	Firmicutes	Mogibacterium	
		Prevotella 7		Senegalimassilia		Coprobacter	Cyanobacteria	Clostridium sp. CAG:306		Vagococcus		GCA-900066755		[Eubacterium] nodatum group	
		Prevotella 9	Bacteroidetes	Odoribacter		Alistipes				Lactococcus		Tyzzerella 3		Sellimonas	
		Prevotellaceae NK3B31 group		Porphyromonas		Alistipes	Firmicutes	Family XIII UCG-001		Christensenellaceae R-7 group		Tyzzerella 3		Tyzzerella 4	
		Alistipes		Prevotella 2		Alistipes		Acetitomaculum		uncultured		Acetanaerobacterium		Tyzzerella	
		gut metagenome		Prevotella 6	Firmicutes	Leuconostoc		Blautia		gut metagenome		Anaerofilum		Fournierella	
	Firmicutes	Lactobacillus		Prevotella		Catabacter		Lachnospiraceae NK4B4 group		Butyrivibrio		Phocea		Faecalicoccus	
		Eubacterium		Prevotella		Christensenellaceae R-7 group		Lachnospiraceae UCG-001		Butyrivibrio		Pseudoflavonifractor		Faecalitalea	
		Finegoldia		Alistipes				Lachnospiraceae UCG-008		Coprococcus 2		Candidatus Stoquefichus	Fusobacteria	Fusobacterium	
		Peptoniphilus		Rikenella		uncultured bacterium		Marvinbryantia		Coprococcus 2		Erysipelatoclostridium	Proteobacteria	Proteus	
		Agathobacter	Firmicutes	Christensenellaceae R-7 group				[Bacteroides] pectinophilus group		Lachnospiraceae ND3007 group		Faecalitalea			
		Blautia		Christensenellaceae R-7 group		Anaerovorax		[Eubacterium] xylanophilum group		Lachnospiraceae UCG-003		Acidaminococcus			
		Blautia		Christensenellaceae R-7 group		[Eubacterium] brachy group		uncultured		[Eubacterium] eligens group		Megamonas			
		Howardella		Christensenellaceae R-7 group		Lachnospiraceae UCG-001		Terrisporobacter		uncultured		Veillonella			
		Lachnoclostridium		Christensenellaceae R-7 group		Sellimonas		Ruminiclostridium 6		Peptococcus	Proteobacteria	Succinivibrio			
		Lachnoclostridium		uncultured		[Eubacterium] fissicatena group		Ruminiclostridium 9		uncultured					
		Lachnospiraceae UCG-010		uncultured Thermoanaerobacterales bacterium		[Eubacterium] ruminantium group		Ruminococcaceae UCG-013		Ruminococcaceae UCG-005					
		[Ruminococcus] torques group		GCA-900066575		Acetanaerobacterium		Ruminococcus 1		Ruminococcaceae UCG-005					
		uncultured		Lachnospiraceae FCS020 group		Anaerofilum		[Eubacterium] coprostanoligenes group		Ruminococcaceae UCG-008					
		uncultured		UC5-1-2E3		Hydrogenoanaerobacterium		Merdibacter		Ruminococcaceae UCG-009					
		uncultured		[Ruminococcus] gauvreauii group		Intestinimonas	Fusobacteria	Leptotrichia		Ruminococcaceae UCG-010					
		Anaerotruncus		Peptococcus		Negativibacillus	Proteobacteria	gut metagenome		Subdoligranulum					
		Flavonifractor		Peptostreptococcus		Ruminococcaceae UCG-002	Tenericutes	gut metagenome		[Eubacterium] coprostanoligenes group					
		Negativibacillus		Anaerotruncus		Ruminococcaceae UCG-007		uncultured Mollicutes bacterium		Catenibacterium					
		Oscillibacter		CAG-352		Ruminococcaceae UCG-010				Phascolarctobacterium					
		Ruminiclostridium 9		Ruminiclostridium 5		Ruminococcus 1			Proteobacteria	Azospirillum sp. 47_25					
		Ruminococcaceae UCG-004		Ruminococcaceae NK4A214 group					Synergistetes	Pyramidobacter					
		Ruminococcaceae UCG-013		Ruminococcaceae UCG-013					Tenericutes	uncultured bacterium					
		[Eubacterium] coprostanoligenes group		Ruminococcaceae UCG-014	Lentisphaerae	Victivallis									
		uncultured		UBA1819	Proteobacteria										
		Holdemania		[Eubacterium] coprostanoligenes group		Sutterella									
		Phascolarctobacterium			Synergistetes	Cloacibacillus									
		Allisonella		Dielma	Tenericutes	uncultured organism									
		Megasphaera	Proteobacteria	Desulfovibrio	Verrucomicrobia	Cerasicoccus									
	Proteobacteria	Sutterella	Tenericutes			uncultured									
			Verrucomicrobia	Akkermansia											

Figure S4

Table 1

Cluster 1		Cluster 2		Cluster 3		Cluster 4		Cluster 5		Cluster 6		Cluster 7		Cluster 8		Cluster 9		Cluster 10	
Phylum	Species	Phylum	Species	Phylum	Species	Phylum	Species	Phylum	Species	Phylum	Species	Phylum	Species	Phylum	Species	Phylum	Species	Phylum	Species
Actinobacteria	Rothia	Euryarchaeota	Methanomassiliicoccus	Firmicutes	Defluviitaleaceae UCG-011	Actinobacteria	Actinomyces	Actinobacteria	Paraeggerthella	Actinobacteria	Slackia	Actinobacteria	Mobiluncus	Euryarchaeota	Methanosphaera	Actinobacteria	Libanicoccus	Bacteroidetes	Prevotella
	uncultured	Actinobacteria	uncultured		Family XIII AD3011 group	Bacteroidetes	gut metagenome	Bacteroidetes	Prevotella 7		uncultured bacterium		Raoultibacter	Actinobacteria	Senegalimassilia		Collinsella	Firmicutes	CHKCI001
Bacteroidetes	Bacteroides	Bacteroidetes	uncultured			Firmicutes	Streptococcus		Alistipes	Bacteroidetes	Butyricimonas		Enterorhabdus				Slackia		Lachnospira
	Coprobacter		Odoribacter		Anaerostipes		Streptococcus	Firmicutes	Gemella		Prevotella	Bacteroidetes	Porphyromonas	Bacteroidetes	Alloprevotella	Bacteroidetes			Lachnospiraceae UCG-001
	Coprobacter	Firmicutes	Christensenellaceae R-7 group		Cuneatibacter				Anaerovorax		Prevotellaceae NK3B31 group		Prevotella		Paraprevotella		Alloprevotella		Lachnospiraceae UCG-010
	Alistipes		Christensenellaceae R-7 group		Eisenbergiella		Christensenellaceae R-7 group		Sellimonas		Prevotellaceae NK3B31 group		Alistipes	Firmicutes	Christensenellaceae R-7 group		Prevotella 2		Roseburia
Cyanobacteria	uncultured bacterium		Eubacterium		Lachnoclostridium		Family XIII AD3011 group		Terrisporobacter		uncultured	Firmicutes	Bacillus		Anaerofustis		Prevotella 2		[Ruminococcus] torques group
Firmicutes	Staphylococcus		Peptoniphilus		Marvinbryantia		Mogibacterium		Ruminococcaceae UCG-009	Epsilonbacteraeota	Campylobacter		Leuconostoc		Eubacterium		uncultured bacterium		uncultured
	Enterococcus		[Eubacterium] brachy group		[Eubacterium] fissicatena group		Agathobacter		Merdibacter	Firmicutes	Lactococcus		Weissella		Howardella	Firmicutes	Lachnoclostridium	Fusobacteria	Leptotrichia
	Christensenellaceae R-7 group		Lachnospiraceae UCG-008		[Eubacterium] hallii group		Lachnoclostridium		Turicibacter		uncultured		Christensenellaceae R-7 group		Peptococcus		Lachnospiraceae FCS020 group		
	Christensenellaceae R-7 group		Tyzzerella 4		[Eubacterium] ventriosum group		Lachnospiraceae UCG-001		Phascolarctobacterium		Ezakiella		Peptoniphilus		Peptococcus		Paeniclostridium		
	Christensenellaceae R-7 group		[Eubacterium] ruminantium group		[Eubacterium] xylanophilum group		Tyzzerella				Murdochiella		Mogibacterium		Ruminococcaceae UCG-005		Paraclostridium		
			CAG-352		[Ruminococcus] gauvreauii group		Anaerotruncus				Family XIII AD3011 group		Coprococcus 3		UBA1819		Romboutsia		
	gut metagenome		Intestinimonas		GCA-900066225		Pseudoflavonifractor				S5-A14a		Sellimonas		[Eubacterium] coprostanoligenes group		[Eubacterium] coprostanoligenes group		
	uncultured Thermoanaerobacterales bacterium		Oscillospira		GCA-900066225		Ruminiclostridium 5				GCA-900066575		uncultured		Catenibacterium		Faecalitalea		
	Anaerofustis		Ruminiclostridium 1		Hydrogenoanaerobacterium		Ruminiclostridium 5				uncultured		Acetanaerobacterium	Proteobacteria	Desulfovibrio		Phascolarctobacterium		
	Family XIII AD3011 group		Ruminiclostridium 6		Intestinimonas		Ruminococcaceae UCG-005				Intestinibacter		Anaerofilum		Sutterella		Allisonella		
	Anaerostipes		Ruminococcaceae NK4A214 group		Intestinimonas		Ruminococcaceae UCG-009				Phocea		Fournierella		Pseudomonas	Proteobacteria	Sutterella		
	Coprococcus 2		Ruminococcaceae UCG-009		Oscillibacter		[Eubacterium] coprostanoligenes group				Ruminiclostridium 9		Oscillibacter						
	GCA-900066575		Candidatus Stoquefichus		Ruminococcaceae NK4A214 group		uncultured				[Eubacterium] coprostanoligenes group		Oscillibacter						
	Lachnoclostridium				Ruminococcaceae UCG-010		Holdemanella				Coprobacillus		Ruminiclostridium 9						
	Lachnospira				Ruminococcaceae UCG-013		Holdemania				Faecalitalea		Ruminococcaceae UCG-004						
	[Ruminococcus] gauvreauii group	Proteobacteria			[Eubacterium] coprostanoligenes group		Veillonella				[Clostridium] innocuum group		Ruminococcaceae UCG-010						
	uncultured		Desulfovibrio				Veillonella				Acidaminococcus		Ruminococcaceae UCG-013						
	uncultured				Candidatus Stoquefichus					Proteobacteria	Parasutterella		Ruminococcus 1						
	uncultured				Dielma						Haemophilus		Solobacterium						
	Romboutsia				uncultured							Fusobacteria	Fusobacterium						
	Candidatus Soleaferrea											Proteobacteria	Sutterella						
	Ruminiclostridium 1																		
	Ruminiclostridium 5																		
	Ruminococcaceae UCG-013																		
	[Eubacterium] coprostanoligenes group																		
	uncultured																		
	Erysipelotrichaceae UCG-003																		
	Phascolarctobacterium																		
Lentisphaerae	Victivallis																		
Proteobacteria	gut metagenome																		
Tenericutes	uncultured organism																		
Verrucomicrobia	Akkermansia																		
